# Midterm outcome after surgical correction of anomalous left coronary artery from the pulmonary artery

**DOI:** 10.1186/s13019-016-0535-7

**Published:** 2016-08-26

**Authors:** Yunfei Ling, Sandeep Bhushan, Qiang Fan, Menglin Tang

**Affiliations:** 1 Department of Cardiovascular Surgery, West China Hospital, Sichuan University, Chengdu, Sichuan People’s Republic of China; 2 Department of Intensive Care Unit, West China Hospital, Sichuan University, No. 37 GuoXue Xiang, Chengdu, Sichuan 610041 People’s Republic of China

**Keywords:** Anomalous coronary artery from pulmonary artery, Outcome, Surgical correction, Coronary artery reimplantation

## Abstract

**Background:**

This study was undertaken to determine the midterm outcome in patients with anomalous left coronary artery from the pulmonary artery (ALCAPA) undergoing coronary reimplantation and Takeuchi repair.

**Methods:**

A retrospective review of patients who had ALCAPA repair between January 2009 and December 2015. Mortality, echocardiography assessment of left ventricular function including ejection fractionand, shortening fraction, severity of mitral regurgitation, stenosis of the coronary ostium were studied retrospectively.

**Results:**

Sixteen patients were described. The mean age at the time of surgery was 22.5 ± 10.3 years (range, 9 months-35.6 years) and 2 patients were younger than 1 year old, Surgical interventions included left coronary artery reimplantation in 13 patients (81 %) and Takeuchi repair in 3 (19 %). Concomitant mitral valve repair was performed in 2 cases, no cases required mechanical circulatory support postoperatively. There was no mortality. At median follow-up of 4.6 years, EF improved from 33.2 % ±6.8 % to 60.9 % ± 8.1 % (*p* <0.05), mean SF from 28.5 % ± 12.1 % to 40.2 % ± 5.4 % (*p* <0.05). Only one patient was with moderate mitral regurgitation. All 16 cases had normal ejection fraction and shortening fraction without stenosis of the coronary ostium at last follow-up.

**Conclusions:**

Early establishment of a 2-coronary artery achieved excellent outcomes without morbidity and mechanical circulatory support. Normal ejection fraction and shortening fraction recovered smoothly. There is no stenosis of the coronary ostium at the midterm follow-up.

## Background

Anomalous left coronary artery from the pulmonary artery (ALCAPA), also known as Bland-White-Garland syndrome, is a rare congenital abnormality that affects 1 of every 300,000 live births and accounts for 0.25 %– 0.5 % of all congenital heart defects [1, 2]. There are two types of ALCAPA: the infant type and the adult type, each of which has different manifestations and outcomes. If left untreated, about 90 % of patients of infant type die within the 1st year of life [3]. However, some adult type patients do not present with symptoms until later in life. These older patients often manifest their anomalies as mitral regurgitation (MR), ischemic cardiomyopathy, malignant dysrhythmias, or even sudden death [4, 5]. So a diagnosis of ALCAPA indicates immediate surgical intervention regardless of the age level except for the newborn.

**Fig. 1:**
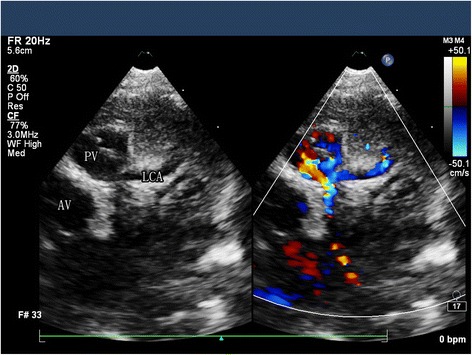
ALCAPA in an infant: TTE showed the LCA originating from the pulmonary artery

**Fig. 2:**
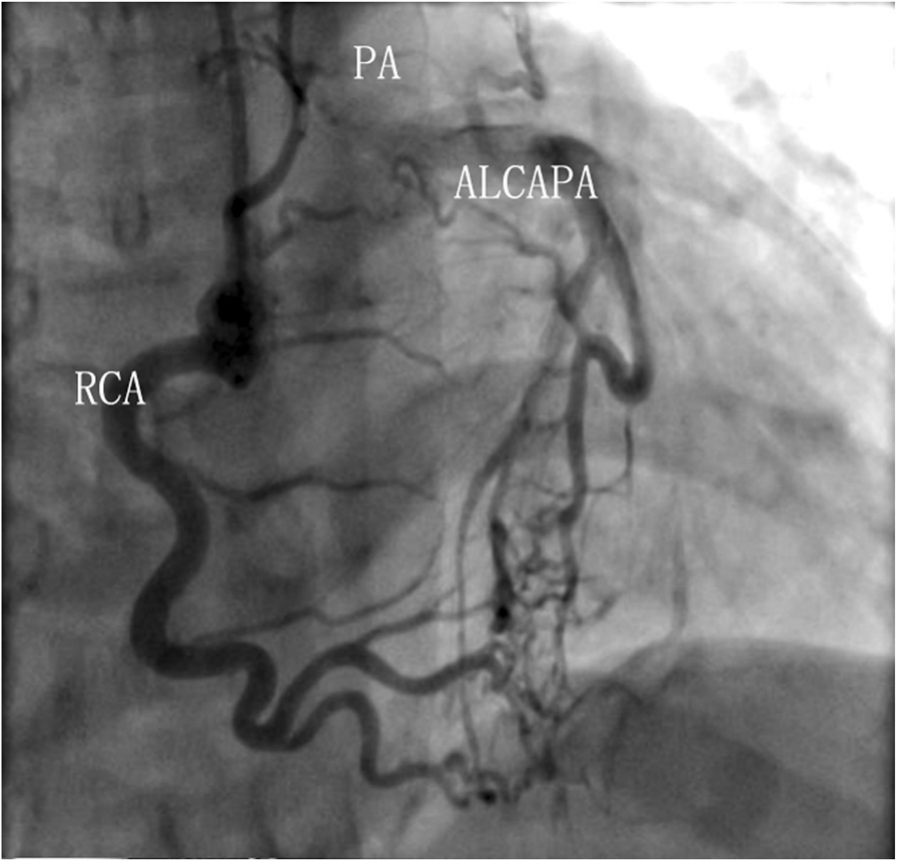
ALCAPA in an adult before the surgery: Coronariograms revealed a tortuous and dilated RCA as well as an equally tortuous and dilated LCA and well-established collateral vessels between LCA and RCA

**Fig. 3:**
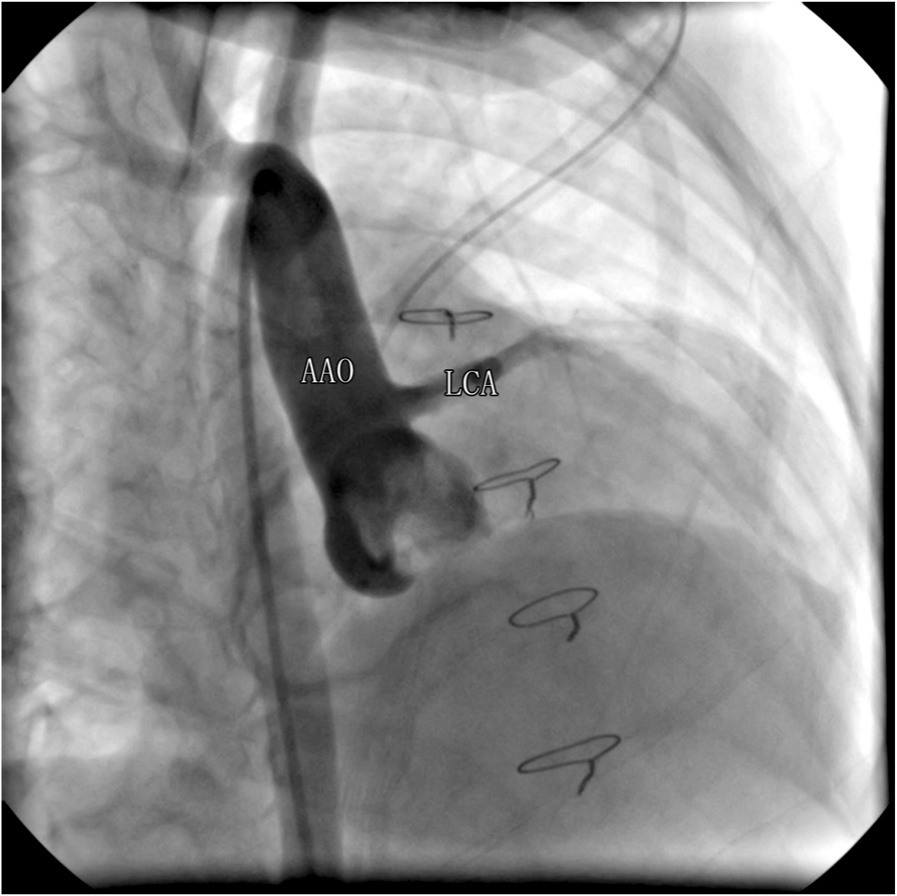
After the surgical correction of ALCAPA. Coronariograms revealed LCA arising from the AAO and well-established collateral vessels between LCA and RCA

Multiple techniques have been introduced to establish a 2-coronary artery system artery, including coronary artery bypass grafting, coronary baffling procedures, and direct reimplantation of the left coronary artery (LCA) to the aorta. Although early diagnosis and prompt surgical intervention lead to excellent results, the possibility of postoperative complications such as persistent MR, late-onset congestive heart failure, and arterial stenosis necessitates long term follow-up. We herein present 16 patients with ALCAPA and discuss the midterm outcome in patients with ALCAPA undergoing a primary LCA reimplantation and Takeuchi repair.

## Methods

### Patients

A retrospective review of charts for patients who underwent surgery for ALCAPA at West China Hospital from January 2009 and December 2015 was performed. Patients whose primary ALCAPA repair was performed at another hospital or whose ALCAPA repair was not the primary operation were excluded. Date included patient demographics, preoperative clinical data, early and late complications, reoperations, and clinical assessment at most recent cardiology follow-up were obtained from electronic medical records and archived paper charts.

Demographic information including age, weight, and BSA were recorded. Operative variables analyzed include mitral valve intervention, duration of mechanical ventilation, duration of intensive care unit and hospital stay and postoperative complications. Ventricular function was assessed by standard echocardiographic methods: ejection fraction (EF) and shortening fraction (SF) on most recent transthoracic echocardiogram. Degree of MR was assessed by qualified and experienced echocardiographic reviewers as none (or trivial), mild, moderate, or severe. Stenosis of coronary ostium was determined by color doppler echocardiography, CT scan or coronary angiogram.

### Surgical technique

Takeuchi repair: After initiation of cardiopulmonary bypass and cardiac arrest, a pulmonary arteriotomy was performed, creating a transverse flap of pulmonary artery tissue. An aortopulmonary window was created, and the pulmonary artery flap was used to baffle the left coronary artery into the aorta. The pulmonary artery was then reconstructed with autologous pericardium [5–7].

LCA reimplantation: To the patients with the origin of the LCA close to the ascending aorta. After standard cardiopulmonary bypass and institution of cardioplegia, the left coronary artery was harvested with a large button of pulmonary arterial wall and widely mobilized without injuring any branches. After inspection of the aortic valve (usually through a separate aortotomy), an aortic flap was performed to minimize torsion of the vessel. The coronary button was anastomosed to the aortic wall with fine polypropylene suture. The pulmonary arterial trunk was reconstructed with a patch of autologous pericardium. However, to the patients with the origin of the LCA far away from the aortic root the coronary ostium was excised along with a strip of the pulmonary artery wall. Autologous pericardium was used to reconstruct the posterior wall of this “elongated” coronary artery and the neo-ostium was then anastomosed end-to-side with the ascending aorta [5, 8, 9].

### Statistically analysis

Continuous variables were reported as medians with minimum and maximum or means with standard deviations. Categoric variables were reported as frequencies with percentages. Independent continuous variables were compared by unpaired Student’s t test for normally distributed data, and Mann–Whitney U-test was used for the comparison of parameters that did not exhibit a normal distribution. Two-tailed *p* value less than 0.05 was considered statistically significant.

## Results

### Perioperative data and postoperative course

A total of 16 patients (62.5 % female) were identified with the diagnosis of ALCAPA (Figs. 1 and 2) and LCA reimplantation was performed in 13 patients (81 %), 3 patients (19 %) was underwent Takeuchi repair. Median age at time of repair was 22.5 ± 10.3 years (range, 9 months-35.6 years) and 2 patients were younger than 1 year old. 2 patients (12.5 %) were associated severe MR, 10 patients (62.5 %) with mild or moderate MR and 4 patients (25 %) without MR. Concomitant mitral valve repair was performed in 2 of 16 patients with significant (severe) preoperative MR. Of the 10 patients with mild or moderate MR who did not undergo surgical intervention for MR, Only 1 patient continued to have moderate MR at 3-year follow-up. No patient required mechanical circulatory support preoperatively or postoperatively (Table 1).

**Table 1:** Characteristics of patients who underwent surgical repair of ALCAPA

Characteristics	*N* = 16
Age, years	22.3 (9 months-35.6 years)
>5 year-old	3 (18.76)
<5 year-old	13 (81.25)
Male	6 (37.5)
Body surface area	0.9 (0.72–1.56)
Surgery type	
Reimplantation	13 (81.25)
Takeuchi repair	3 (18.75)
MR	
Severe	2 (12.5)
Mild or moderate	10 (62.5)
None	4 (25)
Cardiopulmonary bypass time, minutes	154 ± 43
Aortic cross-clamp time, minutes	86 ± 37

### Outcomes of ALCAPA repair at follow-up

Median follow-up time was 4.6 years (range, 1 to 6). Cardiovascular complications at follow-up occurred in 2 patients (%), Right bundle branch block in 1 patient (9 %) and the other who accepted Takeuchi repair was with mild supravalvular pulmonary stenosis which requires further follow-up. There was no death or important morbidities and no stenosis of the coronary ostium (Fig. 3). During the last follow up, only 1patient was associated with moderate MR, The rest were with mild or none MR (Table 2). EF improved from 33.2 % ±6.8 % to 60.9 % ± 8.1 % (*p* <0.05), mean SF from 28.5 % ± 12.1 % to 40.2 % ± 5.4 % (*p* <0.05).

**Table 2:** Middle outcomes of patients who underwent repair of ALCAPA

Outcomes	*n* = 16 (%)
Mechanical ventilation, days	3 (1–10)
ICU length of stay, days	5.4 (2–12)
Hospital length of stay, days	14.6 (11–23)
Cardiovascular complications	
Arrhythmias	1 (6.25)
Supravalvular pulmonary stenosis	1 (6.25)
MR at follow-up	
Severe	0 (0)
Moderate	1 (6.25)
None or mild	15 (93.75)

## Discussion

The origin of the LCA from the pulmonary artery is well tolerated in fetal and early neonatal life because pulmonary arterial pressure is same as systemic pressure, which leads to antegrade flow in both the anomalous LCA and the normal right coronary artery RCA. Soon after birth, when pulmonary arterial pressure decreases, flow in the LCA decreases and then reverses, which leads to myocardial ischemia and infarction [10, 11]. The extent of myocardial necrosis of the left ventricle is determined by the balance between timing of closure of the ductus arteriosus, changes in pulmonary vascular resistance, and speed of development of preexisting collateral circulation between the right and left coronary arteries [12]. Our study demonstrates that excellent early and midterm outcomes with no mortality can be obtained with the contemporary repair of ALCAPA in the majority of patients. Normal systolic function and shortening fraction is recoverable in most patients after establishment of a 2-coronary artery system with a low incidence of reoperations.

Intervening on the mitral valve during the initial surgical repair of ALCAPA remains controversial [13, 14]. Mitral valve intervention was performed in only 2 patients with severe MR, and no other patient has required MR intervention during the surgical repair of ALCAPA. In general, mitral valve repair or replacement is not necessary at the time of ALCAPA repair, especially in the patients younger than 1 year old, but if MR remains persistent, and depending on the severity, it can be managed surgically at a later date and mitral valvuloplasty was preferred [15, 16]. The degree of MR tends to improve with the majority of patients after surgical repair of ALCAPA [17].

The most popular surgical methods are creation of a two-coronary artery system via LCA ligation plus CABG, Takeuchi operation, and LCA reimplantation. Simple ligation of the ACAPA plus CABG, resulting in a single coronary artery system, has been abandoned because of subendocardial ischemia, angina, and sudden death during the follow up [1, 18]. The Takeuchi operation can be adopted when a distance exists between the LCA ostium and the aorta. Its major complications are supravalvular pulmonary stenosis, aortic valve insufficiency, baffle obstruction, and leaks [19]. Most cardiac surgeons prefer to reimplant the anomalous LCA directly onto the aorta and we have modified our technique by using the autologous pericardium to reconstruct the posterior wall of this “elongated” coronary artery and the neo-ostium was then anastomosed end-to-side with the ascending aorta. In our series, 3 patients survived Takeuchi operation and one experienced supravalvular pulmonary stenosis, 13 patients undergoing coronary translocation and achieved excellent results.

## Conclusions

In conclusion, we have achieved excellent outcomes of ALCAPA repaired by the establishment of a two-coronary system by coronary artery reimplantation and Takeuchi repair. Reintervention after ALCAPA repair is rare. Most patients with ALCAPA will have some degree of MR. However, even without intervention upon the mitral valve at time of ALCAPA repair, the regurgitation tends to improve with the majority of patients. Normal EF and SF recovered smoothly. There is no stenosis of the coronary ostium at the midterm follow-up.

## Abbreviations

ALCAPA: Anomalous left coronary artery from the pulmonary artery
EF: Ejection fraction
LCA: Left coronary artery
MR: Mitral regurgitation
RCA: Right coronary artery
SF: Shortening fraction
